# Bioinformatics-based identification of miR-542-5p as a predictive biomarker in breast cancer therapy

**DOI:** 10.1186/s41065-018-0055-7

**Published:** 2018-01-15

**Authors:** Qiong-Ni Zhu, Helen Renaud, Ying Guo

**Affiliations:** 1 Department of Clinical Pharmacology, Xiangya Hospital, Central South University, Changsha, 410008 People’s Republic of China; 2 Institute of Clinical Pharmacology, Central South University, Changsha, 410078 People’s Republic of China; 3 Hunan Key Laboratory of Pharmacogenetics, Changsha, 410078 People’s Republic of China; 4 University of Kansas Medical Center, Kansas City, KS 66160 USA

**Keywords:** Breast cancer, Tamoxifen resistance, Bioinformatics, miRNA-542-5p

## Abstract

**Background:**

Tamoxifen is the first-line hormone therapy for estrogen receptor alpha positive (ERα+) breast cancer. However, about 40% of patients with ERα + breast cancer who receive tamoxifen therapy eventually develop resistance resulting in a poor prognosis. The aim of this study was to mine available data sets in the Gene Expression Omnibus (GEO) database, including in vitro (cell lines) and in vivo (tissue samples), and to identify all miRNAs associated with tamoxifen resistance (TamR) in breast cancer. Secondly, this study aimed to predict the key gene regulatory networks of newly found TamR-related miRNAs and evaluate the potential role of the miRNAs and targets as potential prognosis biomarkers for breast cancer patients.

**Result:**

Microarray data sets from two different studies were used from the GEO database: 1. GSE66607: miRNA of MCF-7 TamR cells; 2. GSE37405: TamR tissues. Differentially expressed microRNAs (miRNAs) were identified in both data sets and 5 differentially expressed miRNAs were found to overlap between the two data sets. Profiles of GSE37405 and data from the Kaplan-Meier Plotter Database (KMPD) along with Gene Expression Profiling Interactive Analysis (GEPIA) were used to reveal the relationship between these 5 miRNAs and overall survival. The results showed that has-miR-542-5p was the only miRNA associated with overall survival of ERα + breast cancer patients who received adjuvant tamoxifen. Targets of has-miR-542-5p were predicted by miRanda and TargetScan, and the mRNA expression of the three 3 target gene, Tyrosine 3-Monooxygenase/Tryptophan 5-Monooxygenase Activation Protein Beta (YWHAB), Lymphocyte Antigen 9 (LY9), and Secreted Frizzled Related Protein 1 (SFRP1) were associated with overall survival in 2 different databases. Copy-number alterations (CNAs) of SFRP1 confer survival disadvantage to breast cancer patients and alter the mRNA expression of SFRP1 in cBioPortal database.

**Conclusion:**

This study indicates that miRNA has-miR-542-5p is associated with TamR and can predict prognosis of breast cancer patients. Furthermore, has-miR-542-5p may be acting through a mechanism involving the target genes YWHAB, LY9, and SFRP1. Overall, has-miR-542-5p is a predictive biomarker and potential target for therapy of breast cancer patients.

**Electronic supplementary material:**

The online version of this article (10.1186/s41065-018-0055-7) contains supplementary material, which is available to authorized users.

## Background

Breast cancer is the most common malignancy among women and second leading cause of cancer death in the USA [1–3]. Tamoxifen is a selective estrogen receptor modulator and one of the most effective adjuvant treatments for estrogen receptor alpha positive (ERα+) breast cancer patients in clinical practice. Unfortunately, about 40% of breast cancer patients with ERα + who receive tamoxifen therapy develop tamoxifen resistance (TamR) [4, 5].

The mechanisms of TamR include aberrant ERα expression, altered signal transduction pathways, imbalance of co-regulatory proteins, genetic polymorphisms in tamoxifen metabolism [6, 7], and epigenetic modifications including expression changes of microRNAs (miRNAs) [8]. miRNAs are a class of small non-coding RNAs that post-transcriptionally control the translation and stability of mRNAs [9] by forming specific base-pairing interactions between the 5′ end of the miRNA (seed region) and the miRNA response elements within the coding region or untranslated regions (UTRs) of mRNAs, leading to mRNA destabilization and/or translational inhibition [10]. Fundamentally, miRNAs provide an extra level of regulation to gene expression.

The role of miRNAs in the progression of endocrine-resistant breast cancer is of intense interest as they are promising biomarkers and therapeutic targets to counter metastatic disease [11]. Consequently, studies are rapidly emerging on the role of miRNAs in endocrine resistant breast cancer [12, 13]. Several miRNAs have been associated with TamR in MCF-7 cells exposed to tamoxifen (1 μM) for >12 months [14]. For example, expressions of miR-190b and miR-516a-5p were altered in TamR cells and were predictive of treatment outcome in a cohort of ERα + breast cancer patients receiving adjuvant tamoxifen mono-therapy [13]; miR-519a confers TamR by targeting a network of tumor-suppressor genes in ERα + breast cancer [15]; miR-27b is epigenetically downregulated in TamR breast cancer cells due to promoter methylation and also regulates tamoxifen sensitivity by targeting High Mobility Group Box 3 [16]; miR-320a sensitizes TamR breast cancer cells to tamoxifen by targeting CAMP Regulated Phosphoprotein 19 and ERRγ in breast cancer cells and tissues [17]; and miR-378a-3p regulates tamoxifen sensitivity in MCF-7 cells through targeting Golgi Transport 1A [18].

In this study, we used a meta-analysis approach to discover which miRNAs are associated with TamR across multiple studies in order to reveal strong miRNA candidates and down-stream target genes that can be used as predictive biomarkers and novel targets for therapy. To accomplish this, we mined the Gene Expression Omnibus (GEO) database and analyzed all the available in vitro (cell lines) and in vivo (tissue samples) data sets for miRNAs associated with TamR in breast cancer, identified all the miRNAs related to TamR, predicted the key gene regulatory network of a novel miRNA associated with TamR, and evaluated the potential role of this miRNA and target genes as prognosis biomarkers for breast cancer patients. This is the first study to indicate and validate the potential association of miR-542-5p and its downstream genes Tyrosine 3-Monooxygenase/Tryptophan 5-Monooxygenase Activation Protein Beta (YWHAB), Lymphocyte Antigen 9 (LY9), and Secreted Frizzled Related Protein 1 (SFRP1) with the prognosis of breast cancer.

## Methods

### Microarray data

From the GEO database we searched all available data on tamoxifen treated samples of breast cancer tissue (with resistance status) and all TamR breast cancer cell samples (Additional file 1), and found two sets of miRNA microarrays: GSE66607 and GSE37405. GSE66607 employed an Affymetrix GeneChip® miRNA 3.0 microarray to identify differentially expressed miRNAs between tamoxifen sensitive MCF7 parent cells and tamoxifen-induced resistant cells. The dataset of GSE37405 employed an Exiqon mercury LNA microRNA array on 152 ERα + primary tumors from high-risk breast cancer patients, all patients had received adjuvant tamoxifen as mono-therapy (median clinical follow-up: 4.6 years). Patients were divided into two groups, patients without recurrence (denoted N *n* = 76), and recurrence (denoted R n = 76) [12].

### Quality control

To ensure accuracy and reliability of this experimental design, the median value of NUSE (Normalized Unscaled Standard Errors) and the median value of RLE (Relative Log Expression) were used as evaluation criteria for each microarray. This method has been previously published [19].

### Identification of differentially expressed genes

In GSE66607, differentially expressed genes were analyzed by Gene Cloud of Biotechnology Information (GCBI) working platform (GMINIX Informatics Ltd. Co, Shanghai, P. R. China), the rank is sorted by the absolute value of the fold change. Differentially expressed genes in GSE37405 were analyzed using GEO2R, an R-based web application that helps users analyze GEO data [20]. Data points with a *p*-value <0.05 were retained (https://www.ncbi.nlm.nih.gov/geo/geo2r/?acc=GSE37405). All data are displayed as log2 transformed.

### Strategy

**Fig. 1:**
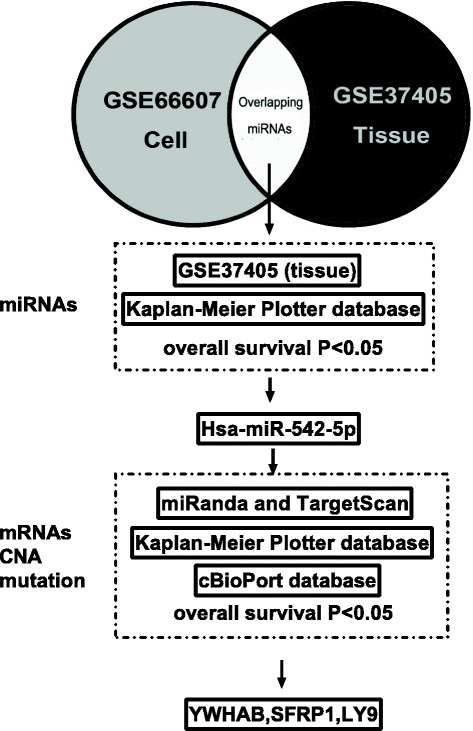
The multi-step strategy used in this study. First, significantly changed miRNAs in GSE66607 (cells for miRNA in MCF-7/TamR vs MCF-7) and GSE37405 (tissues for breast cancer tissues) were identified. Second, GSE37405 and Kaplan-Meier Plotter Database (KMPD) for miRpower of breast cancer as the validated set were used to screen overlapped miRNAs in step 1 that related to overall survival. Third, for the target mRNA genes, KMPD, and Gene Expression Profiling Interactive Analysis (GEPIA), were used to reveal the relationship between target genes and overall survival

As shown in Fig. 1 we used a multi-step strategy to identify miRNAs that become differentially expressed in TamR breast cancer cells and tissues. First, significantly changed miRNAs in GSE66607 (cells) and GSE37405 (tissues) were identified. Second, GSE37405 and Kaplan-Meier Plotter Database (KMPD) - an online survival analysis tool to rapidly assess the effect of 22,277 genes on breast cancer prognosis using microarray data of 1809 patients who accept endocrine therapy [21] with miRpower of breast cancer as the validated set [21] were used to screen overlapped miRNAs in step 1 that related to overall survival. Third, Targets of miRNAs were predicted by miRanda and TargetScan, to reveal the relationship between miRNA target genes and overall survival, the following tools and databases were used: KMPD, GEO (Affymetrix HGU133A and HGU133 + 2 microarrays) [22], and Gene Analysis Differential genes analysis (GEPIA). GEPIA is a newly developed interactive web server using a standard processing pipeline for analyzing the RNA sequencing expression data of 9736 tumors and 8587 normal samples in the Cancer Genome Atlas (TCGA) and the Genotype-Tissue Expression projects [23]. In addition, Molecular Taxonomy of Breast Cancer International Consortium (METABRIC) and TCGA Provisional data in cBioPortal [24] were used to investigate the relationship between mutation/ Copy-number alterations (CNAs) and patients overall survival as well as the mRNA expression of target genes in breast cancer.

### Statistical analysis

For each triplicate of microarray data, analysis of variance for comparisons between more than two groups was used in analyzing the CNAs and mRNA expression. A *p*-value of <0.05 was considered statistically significant. Differences in dichotomous variables between groups were compared using Pearson’s chi-square statistic or Fisher’s exact test. Differences in continuous variables between groups were compared using the median score test. Kaplan-Meier overall survival curves were computed and compared using the log-rank statistic. Adjusted *p*-values were computed using Sidak’s procedure for pairwise comparisons after a significant log-rank test in overall survival value.

## Result

### Identification of differentially expressed genes

**Fig. 2:**
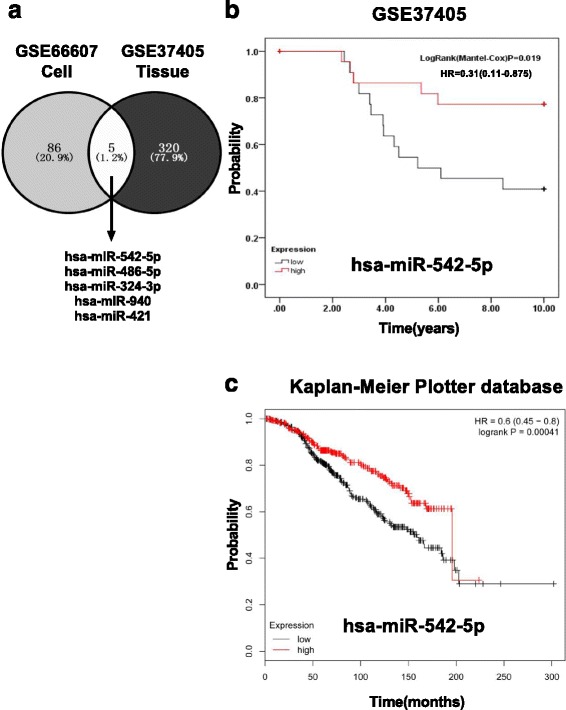
Survival of tamoxifen resistance specific markers. **a**. The two Gene Expression Omnibus (GEO) sets (GSE66607 and GSE37405) were overlapped and 5 miRNAs (Fig. 2a, hsa-miR-324-3p, hsa-miR-486-5p, hsa-miR-940, hsamiR-542-5p, hsa-miR-421) were obtained. **b**. Association of has-miR-542-5p expression with tamoxifen resistance outcome in GSE37405. Survival curves were generated using the Kaplan-Meier method and the log rank test was used to assess the statistical significance of differences. (Log Rank =0.019), Red: high expression; black: low expression. **c**. In the Molecular Taxonomy of Breast Cancer International Consortium (METABRIC) data set, the high expression of has-miR-542-5p was also associated with overall survival in patients with ER- positive endocrine therapy in 695 patients (logrankP = 0.00041, HR = 0.6(0.45–0.8). Red: high expression; black: low expression

There were 91 differentially expressed genes (48 up-regulated and 43 down-regulated miRNA genes) between normal and TamR MCF-7 cells in GSE66607 (Additional file 2). In GSE37405, 325 miRNAs were substituted as differentially expressed genes (Additional file 3). The two differentially expressed gene sets were overlapped and 5 miRNAs (Fig. 2a, hsa-miR-324-3p, hsa-miR-486-5p, hsa-miR-940, hsa-miR-542-5p, hsa-miR-421) were identified(Additional file 4).

### Survival analysis of TamR specific markers

Kaplan-Meier analysis of GSE37405 showed that high expression of has-miR-542-5p in TamR breast cancer tissues conferred a survival advantage to patients with tamoxifen systemic treatment [logRank (Mantel-Cox) *p* = 0.019, HR = 0.31(0.11–0.875), Fig. 2b]. In the METABRIC data set of KMPD, the high expression of has-miR-542-5p was also associated with overall survival in 695 patients with ERα + breast cancer receiving endocrine therapy [logrankP = 0.00041, HR = 0.6 (0.45–0.8), Fig. 2c]. Expression of the other 4 TamR-related miRNAs was not associated with overall survival in the GSE37405 dataset, but 2 of them [hsa-miR-486-5p: logrankP = 0.04900, HR = 0.74 (0.55–1); hsa-miR-421: logrankP = 0.05800, HR = 1.33 (0.99–1.8)] seemed to be conferred a survival relationship to patients with tamoxifen systemic treatment in the KMPD dataset (Additional file 4).

### miRNA target gene prediction and overall survival

#### 1 miRNA target prediction

**Fig. 3:**
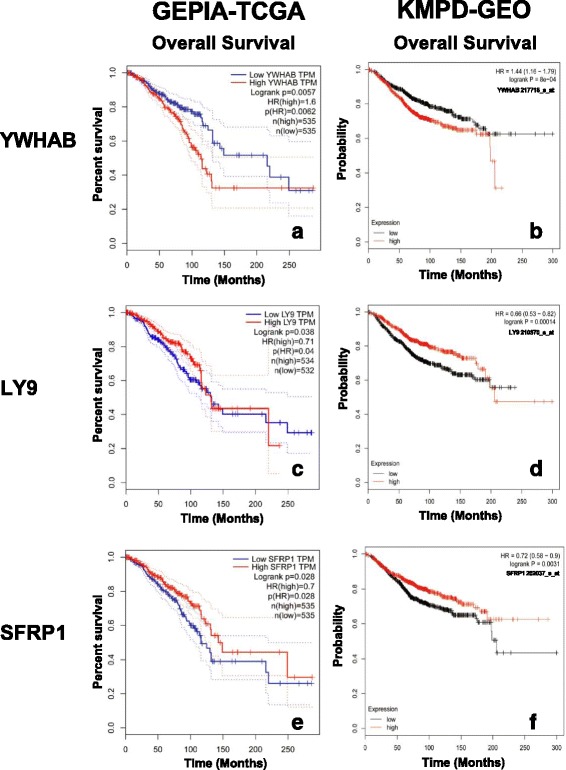
The mRNA expression of has-miR-542-5p target genes and overall survival value. The mRNA expression of has-miR-542-5p target genes including Tyrosine 3-Monooxygenase/Tryptophan 5-Monooxygenase Activation Protein Beta (YWHAB), Lymphocyte Antigen 9 (LY9), and Secreted Frizzled Related Protein 1 (SFRP1) associated with overall survival in breast cancer. The left panel (YWHAB **a**, LY9 **c**, SFRP1 **e**) were verified in Gene Expression Profiling Interactive Analysis (GEPIA) database Red: high expression; blue: low expression; and the right panel (YWHAB **b**, LY9 **d**, SFRP1 **f**) results comes from KMPD. Red: high expression; black: low expression

Since miRNAs negatively regulate gene expression, upregulated miRNAs may result in downregulated target mRNAs. Targets of miRNAs were predicted by miRanda and TargetScan(Additional file 5). A total of three has-miR-542-5p target mRNAs were closely related to overall survival (Fig. 3) of breast cancer patients in both KMPD and GEPIA, and these are: YWHAB, LY9, and SFRP1.

#### 2 survival analysis of target genes

High expression of YWHAB confers a survival disadvantage to breast cancer patients in both GEPIA with TCGA samples [logRank *p* = 0.0057, HR (high) =1.6, Fig. 3a] and KMPD with GEO samples [217718_s_at, logRank *p* = 8e-04, HR = 1.44(1.16–1.79), Fig. 3b]. While high expression of the other two target genes LY9 [GEPIA: logrankp = 0.038, HR (high) = 0.71, Fig. 3c; KMPD: 210370_s_at, logRank*p* = 0.00014, HR = 0.66(0.53–0.82), Fig. 3d] and SFRP1 [GEPIA: logrankP = 0.028, HR(high) = 0.7, Fig. 3e; KMPD: 202037_s_at, logRank *p* = 0.00031, HR = 0.72(0.58–0.9), Fig. 3f] conferred a survival advantage to breast cancer patients.

**Fig. 4:**
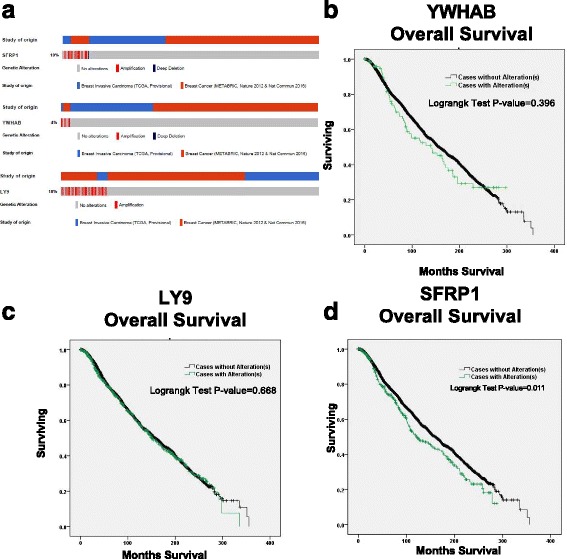
Copy-number alterations of has-miR-542-5p target genes and overall survival value. Has-miR-542-5p target genes expression associated with copy-number alterations in breast cancer. **a**: The overall alteration situation of the 3 genes. **b**-**d**: The relationship between Copy-number alterations (CNAs) and overall survival value verified in cBioPortal (TCGA and Metabric) database (**b** for YWHAB, **c** for LY9, **d** for SFRP1) black: case without alterations; green: case with alterations

**Fig. 5:**
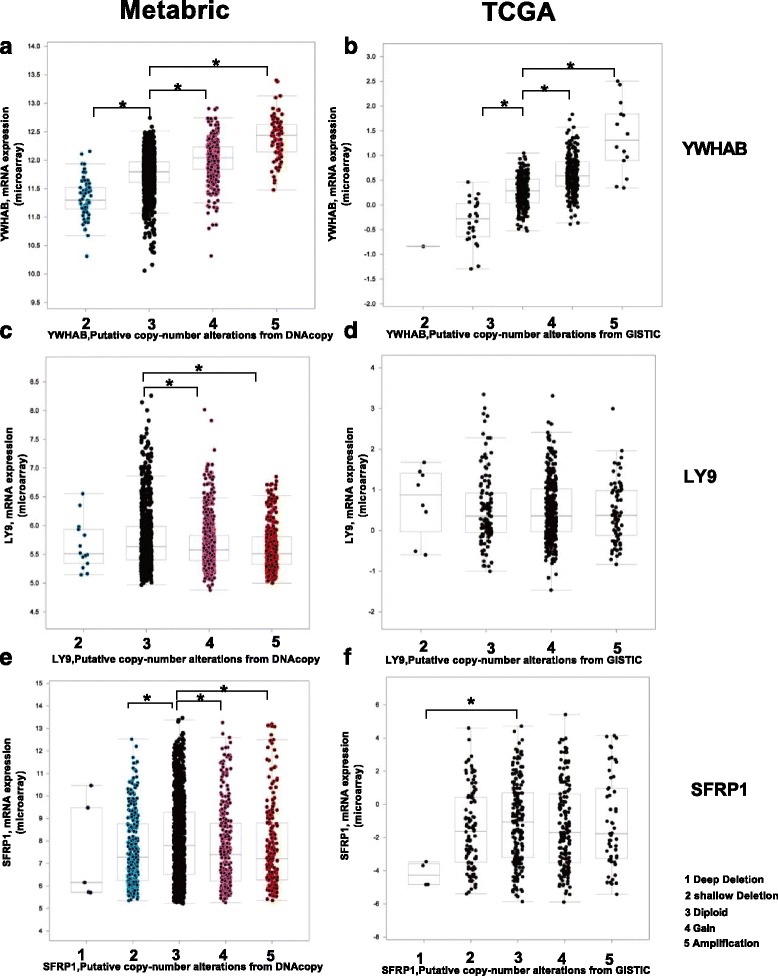
CNAs and mRNA expression of has-miR-542-5p target genes. Has-miR-542-5p target genes expression associated with CNAs in breast cancer. The left panel (**a** for YWHAB, **c** for LY9, **e** for SFRP1) were verified in Metabric database; and the right panel (**b** for YWHAB, **d** for LY9, **f** for SFRP1) results comes from KMPD. These samples were separated in to five categories, 1-Deep deletion (homozygous deletion), 2-Shallow deletion (heterozygous deletion), 3-diploid, 4-Gain (low level amplification), and 5-amplification (high level amplification). *p* < 0.05 compared to diploid group

The cBioPortal was used to identify CNAs associated with survival in YWHAB, LY9 and SFRP1 and the relationship between CNAs and mRNA expression. There are 10% cases had genetic alterations in SFRP1 (Fig. 4a), and these alterations confer survival disadvantage to breast cancer patients in TCGA and METABRIC cohort [Fig. 4d logrank *P* = 0.011, HR = 1.245 (1.051–1.474)]. The mRNA expression of SFRP1 was lower in cases with deep deletion (*p* = 0.502), shallow deletion (p = 0.000), gain (*p* = 0.007) and amplification (*p* = 0.046) than that without change in METABRIC samples (Fig. 5e), which was similar to the results in TCGA (Fig. 5f). Alterations of the other 2 genes were not associated with overall survival in the cBioPortal (and Fig. 4b and c). And the difference between the mRNA expression and CNAs of these 2 genes were not obvious (Fig. 5a and b for YWHAB, c and d for LY9). In addition, the mutations of the predicted genes were also investigated in the cBioPortal, and they are all rare ones and little data is available for further analysis.

## Discussion

In the present study, we mined all miRNA profiling data of breast cancer available in the GEO database to examine miRNA changes associated with TamR, and identified a novel target for TamR diagnosis and prognosis of breast cancer patients. It is interesting to note that we found highly expressed has-miR-542-5p is associated with better overall survival in patients with ERα + breast cancer receiving endocrine therapy. In addition, three targets of has-miR-542-5p (YWHAB, LY9 and SFRP1) were also associated with prognosis in these patients.

Understanding the molecular mechanism of TamR is of critical importance for diagnosis and treatment of breast cancer patients [25, 26]. The results for 4 of the 5 TamR-related miRNAs found in this study corroborate with past findings. For instance, down regulation of miR-27b-3p enhances TamR in breast cancer by increasing NR5A2 and CREB1 expression [27]; deep sequencing analysis of the breast cancer cell line MCF7 over-expressing miR-335-5p and miR-335-3p revealed that this miRNA duplex represses gene expressions of factors involved in the ERα signaling pathway; thus, enhancing TamR in MCF7 cells [28]; miR-29b-1 has tumor suppressor activity in TamR cells [29]; and miR-221/222 is involved in cell cycle deregulation in breast cancer drug resistance [30]. Therefore, the novel strategy used in our study provides a reliable and robust meta-analysis approach to find strong miRNA candidates for prognosis biomarkers or novel therapeutic targets. Moreover, in the current study all the available miRNA data of breast cancer in GEO were included in the analysis and investigated with a comprehensive examination and validation. With this approach we identified a novel and very promising TamR-associated miRNA.

Examining the relationship between TamR-related miRNAs and overall survival using data from GSE37405 and METABRIC, we identified a new candidate factor for prognosis, has-miR-542-5p. Two of the other 4 miRNAs, has-miR-486-5p and has-miR-421, might also be associated with overall survival in ERα + breast cancer patients receiving endocrine therapy. A significant change in their expressions was detected in the larger sample set (Additional file 4); however, there are discrepancies in the expressions of these 2 miRNAs between the GEO and KMPD databases, which might be caused by different sample sizes of the datasets. The role that has-miR-486-5p plays in the prognosis of breast cancer patients has also been indicated in other investigations [31].

Studies have described the function of has-mir-542-5p in cancers. Kaplan-Meier survival analysis revealed significantly reduced event-free survival in patients with low miR-542-5p expression (*p* < 0.001, log-rank test) in neuroblastoma, which is consistent with the results of our study [32, 33]. The difference of transcript/translation expression for has-miR-542-5p between local and metastatic disease is very obvious in neuroblastoma [32], breast cancers [34], and endometrial carcinosarcoma [35]. Our research shows that high expression of has-miR-542-5p confers a survival advantage to patients receiving tamoxifen treatment (Fig. 2b), though has-miR-542-5p is overexpressed in breast cancer with lymph node metastasis [34]. Together, these studies suggest that has-miR-542-5p might be closely associated with epithelial-mesenchymal transition (EMT), a process by which epithelial cells lose their cell polarity and cell-cell adhesion ability, thus gaining migratory and invasive properties. Has-miR-421, another differentially expressed miRNA found in our study to be related to overall survival in the KMPD dataset, has previously been found to inhibit breast cancer metastasis by target E-cadherin [36] or metastasis associated 1 [37]. Recurring tumors after treatment with chemotherapy have an increased proportion of cells expressing EMT-associated genes, thus highlighting the importance of EMT in therapeutic resistance in breast cancer [38]. Other processes might also be involved in TamR. For instance, inhibition of miR-542-5p promoted efficient DNA repair and activated expression of Notch reporters [39]. In another report, miR-542-5p induced double-strand DNA breaks and reactive oxygen species accumulation in transfected cells [40], which might be related to the significantly increased sensitivity of therapy-resistant derivatives of breast cancer cells to a complex of DNA ligase III inhibitors that increased the number of DNA double-strand breaks [41].

MiRNAs affect the development of various diseases by inhibiting the expression of their target mRNAs [42]. Understanding the relevance of miRNA and mRNA expression patterns in TamR breast cancer is important to elucidate the relationship between the pathophysiological process and gene transcription. We analyzed the relationship of the downstream target genes of miR-542-5p with survival of the patients in the KMPD and GEPIA databases, and found 3 target genes (YWHAB, LY9 and SFRP1) associated with overall survival. YWHAB plays a key role in cellular proliferation and oncogenic transformation and has been reported to be involved in the development of breast cancer [43] and the activation of tumor/metastasis pathways and inhibition of apoptosis [44]. Knockdown of YWHAB upregulated connectivity hubs, successfully inhibited in vitro proliferation, colony formation, anchorage independence, migration and invasion in MDA-MB-231 cells [45]. This is consistent with our results that high YWHAB expression is associated with poor overall survival in breast cancer patients (Fig. 3a, b), which could also be explained by low expression of has-mir-542-5p for these patients shown in Fig. 2b, c.

The hazard ratio of the other two target genes (LY9 and SFRP1) could not be explained by negative regulation of miR-542-5p. This may be due to the different mechanism or other regulating factors in breast cancer. LY9 belongs to the signaling lymphocyte activation molecules family of immunomodulatory receptors and interacts with the adaptor molecule signaling lymphocyte activation molecules-associated protein [46], which is involved in hematopoietic stem cell differentiation pathways and lineage-specific markers [47]. Diseases associated with LY9 include Systemic Lupus Erythematosus. To our knowledge, there is no previously published report associating LY9 with breast cancer; thus, further studies are needed to evaluate the role of LY9 in the development of TamR breast cancer. SFRPs act as soluble modulators of Wnt-pathway signaling, which is associated with cancer proliferation, migration and invasion of breast cancer cells. Studies have reported that miR-27a affects in vivo and in vitro proliferation, migration, and invasion of breast cancer cells through targeting the SFRP1 gene via Wnt/β-catenin signaling pathway [48]. Expression of SFRP1 leads to antitumor synergy of combined HDAC and methyltransferase inhibitors in chemo-resistant cancers [49]. Furthermore, SFRP1 DNA methylation was reported to accumulate with age in normal-appearing kidney tissues and be associated with increased renal cancer risk [50], which indicates the importance of SFRP1 in cancer. In the present study, CNAs of SFRP1 gene is also closely related to prognosis (Fig. 4d), which might be related to the decreased mRNA expression in patients with these genetic alterations (Fig. 5 e and f). The mechanism of how has-mir-542-5p regulates TamR in breast cancer and how it influences its downstream target genes warrants further study.

## Conclusion

In conclusion, our study provides a comprehensive bioinformatics analysis of TamR-related miRNAs and reveals the association of certain miRNAs with the overall survival of breast cancer patients. The current study indicates that has-miR-542-5p is related to TamR and affects the prognosis of breast cancer patients. Regulating the target genes YWHAB, LY9 and SFRP1 may be a possible mechanism of has-miR-542-5p in TamR of breast cancer.

## Additional files

Additional file 1: Data sets of tamoxifen resistant breast cancer samples in the GEO. (XLSX 10 kb)

Additional file 2: Identification of DEGs in GSE66607. (XLSX 14 kb)

Additional file 3: Identification of DEGs in GSE 67916. (XLSX 26 kb)

Additional file 4: Rlationship between miRNA and overall survival rate for the overlap miRNAs. (XLSX 9 kb)

Additional file 5: Predicted targets of has-miR-542-5p. (XLSX 32 kb)

## Abbreviations

CNAs: Copy-number alterations
EMT: Epithelial-mesenchymal transition
ERα +: Estrogen receptor alpha positive
GCBI: Gene Cloud of Biotechnology Information
GEO: Gene Expression Omnibus
GEPIA: Gene Expression Profiling Interactive Analysis
KMPD: Kaplan-Meier Plotter Database
LY9: Lymphocyte Antigen 9
METABRIC: Molecular Taxonomy of Breast Cancer International Consortium
MIRNA: MicroRNA
SFRP1: Secreted Frizzled Related Protein 1
TamR: Tamoxifen resistance
TCGA: The Cancer Genome Atlas
UTRs: Untranslated regions
YWHAB: Tyrosine 3-Monooxygenase/Tryptophan 5-Monooxygenase Activation Protein Beta
